# Enhancing Cyber Security of LoRaWAN Gateways under Adversarial Attacks

**DOI:** 10.3390/s22093498

**Published:** 2022-05-04

**Authors:** Ali Mohamed, Franz Wang, Ismail Butun, Junaid Qadir, Robert Lagerström, Paolo Gastaldo, Daniele D. Caviglia

**Affiliations:** 1 Department of Computer Science and Engineering, Chalmers University of Technology, SE-412 96 Gothenburg, Sweden; almoha@student.chalmers.se (A.M.); wfranz@student.chalmers.se (F.W.); 2 Department of Computer Engineering, Konya Food and Agriculture University, Konya 42080, Turkey; 3 Department of Electrical Engineering and Computer Science, KTH Royal University of Technology, SE-100 44 Stockholm, Sweden; junaidq@kth.se or robertl@kth.se (R.L.); 4 Department of Electrical, Electronic and Telecommunications Engineering and Naval Architecture (DITEN), University of Genoa, 16145 Genoa, Italy; paolo.gastaldo@unige.it (P.G.); daniele.caviglia@unige.it (D.D.C.)

**Keywords:** cybersecurity, LoRaWAN, security, vulnerabilities, gateway, attacks, authentication

## Abstract

The Internet of Things (IoT) has disrupted the IT landscape drastically, and Long Range Wide Area Network (LoRaWAN) is one specification that enables these IoT devices to have access to the Internet. Former security analyses have suggested that the gateways in LoRaWAN in their current state are susceptible to a wide variety of malicious attacks, which can be notoriously difficult to mitigate since gateways are seen as obedient relays by design. These attacks, if not addressed, can cause malfunctions and loss of efficiency in the network traffic. As a solution to this unique problem, this paper presents a novel certificate authentication technique that enhances the cyber security of gateways in the LoRaWAN network. The proposed technique considers a public key infrastructure (PKI) solution that considers a two-tier certificate authority (CA) setup, such as a root-CA and intermediate-CA. This solution is promising, as the simulation results validate that about 66.67% of the packets that are arriving from an illegitimate gateway (GW) are discarded in our implemented secure and reliable solution.

## 1. Introduction

The large pool of the Internet of Things (IoT) paradigm enables an extensive research community in industry and academia. IoT devices are being utilized in various applications at the domestic and industrial levels: for instance, they can find applications in machine-to-machine (M2M) communication, tactical surveillance, smart city, and smart grid. IoT devices rely on wireless communication including Bluetooth, ZigBee, WiFi, and NB-IoT [1]. However, such technologies are not able for long-distance communication. Therefore, LoRaWAN [2] was recently introduced as a promising technology that provides communication over long distances with the price of low data rates.

LoRa is a proprietary physical layer protocol developed by Semtech Inc., Camarillo, CA, USA, which provides very long distance communication (20 km) over extremely low power consumption. LoRa makes use of a special modulation known as the Chirp Spread Spectrum (CSS) technique that may offer long-lasting communication (up to 10 years), even for a small battery (1.5V-AA). LoRa-based devices operate on industrial, scientific and medical (ISM) bands to put forward the packet about 2–5 km in and up to 45 km in rural areas [3].

The overall security in LoRaWAN is evolving and technically challenging [4]. It uses end-to-end encryption, with AES 128-bit-key operating in CTR mode; additionally, every message is signed.

There are various papers available in the literature that consider the security vulnerabilities of LoRaWAN. For instance, Yang et al. [5] made a scientific study on security vulnerabilities in LoRaWAN published in 2018, which mentions several different malicious attacks that the LoRaWAN network is susceptible to due to the gateway vulnerability. It has then be verified again in 2018 by Butun et al. [6,7], who conducted a security risk survey of the LoRaWAN-specification and in their work compiled a list of attack vectors that assesses the likelihood and risk associated with each attack vector. In 2019, Eldefrawy et al. [8] further verified the inherent security problems by executing a formal security analysis.

Several security breaches identified in above mentioned are summarized as follows:

- **Man-in-the-Middle (MITM) Attacks:** LoRaWAN is vulnerable to a specific MITM attack called bit-flipping attack, which changes the content of a message between the NS and AS.
- **Network Flooding Attack:** Here, the end device can be captured and made to attack the rest of the network by flooding it with packets.
- **Network Traffic Analysis:** Known as an eavesdropping attack, this is done with a rogue gateway to receive packets and deduce some information of its contents. It would still need a key to decode it, but other information such as the activity in a certain location can be observed.
- **Physical Attack:** Here, the node is physically compromised, either destroyed, stolen or cloned. It is thus of high importance to have adequate protection against firmware change that could lead to the reuse of key material.
- **Radio Frequency (RF) Jamming Attack:** It is possible to jam the reception of a signal in a node, which could be used for more advanced attacks such as a replay attack to be effective.
- **Self-Replay Attack:** An attack that exploits the join procedure by replicating a join request while jamming the original sender. It is thus able to look legitimate until the daily quota of the impersonating ED depletes.

As mentioned above, many authors contributed in this field to improve overall network security of the LoRaWAN, especially seeking remedies to key management and distribution challenges. However, the network has overlooked one critical class of components, which is the gateways.

LoRaWAN implementation is accomplished in five steps such as end devices, gateways, network server (NS), join server (JS), and application server (AS), which are further described in the upcoming section. The gateway constitutes the weakest point in the LoRaWAN implementation. Most of the deployments feature a small number of gateways, and in some cases, just one or two of them run the network. So, an attacker having gateway attacking motivation can capture or physically destroy the gateway, which can significantly affect the successful communication of the end device to the other entities, i.e., network and application server. The network server is dependent on the gateway, and the compromised gateway can falsely lead the packet toward the network server. The gateway cyber security breach is discussed in paper [9], and the authors argued that the attacker can capture the valid data during transmission and then alter or replicate it. The LoRaWAN gateway is susceptible to jamming attacks and results in denial-of-service that disrupts the communication between the node and the gateway.

Having poorly secured gateways is a major flaw that can affects the vulnerability of the rest of the network. A lack of authentication and message authentication (MAC) between GW and the NS makes it possible for an attacker to execute various attacks targeting the network’s availability.

If a gateway is not properly secured with an authentication mechanism, it would be susceptible to a variety of malicious rogue-GW attacks, which could disrupt its availability. This vulnerability could disrupt the traffic and increase collisions of transmitted packets. There could be dire consequence if not one but multiple gateways are either captured or replicated with malicious intent, which can cause disastrous disruptions in the traffic flow and result in the collapse of the network.

This paper presents a novel technique that enhances the cyber security of the LoRaWAN gateway. The proposed technique consists of a public-key infrastructure (PKI) that constitutes a two-tier certificate authority (CA) solution. The two-tier CA solution tackles the single-point failure setup by employing the root-CA and intermediate-CA setup. The simulation results revealed that the proposed technique successfully mitigates malicious attacks such as Selective Forwarding Attacks. For a more detailed presentation of this work, please refer to the thesis by Mohamed and Wang, named “Rogue Gateway Attacks Against LoRaWAN and Their Mitigation” [10].

The rest of the paper is organized as follows. Section 2 discusses related work in the given field. Section 3 describes the network architecture and related basics of LoRaWAN. The design and implementation have been addressed in Section 4. The results from the proposed solution are explained in Section 5 and discussed thoroughly in Section 6. Finally, the overall work is concluded in Section 7, which also provides indications for future improvements.

## 2. Related Work

This section deals with cyber security breaches of LoRaWAN gateways that have been recently introduced in the literature.

Gateways are transparent, and they are not authenticated at any way. Therefore, the authors in [11] stated that rogue gateways can be harmful and introduce Beacon Attacks by bursting false beacons repeatedly. The same authors have presented a scenario of setting up a malicious gateway that captures and drops certain packets. The situation become a worse problem when there is only one gateway in the given range; therefore, the network server may not find any other way to get packets from the end device.

The authors in [12] presented various radio jamming attacks that are associated with cheap hardware with a low capability radio module and a micro-controller. The first attack, such as a triggered jamming attack, happens when the packet is detected passing through the dedicated channel. The selective jamming attack is possible only when there is LoRaWAN packet activity in the channel.

Forcing a class change breach is discussed in [12], where the authors stressed that when the gateway refrains from sending beacon messages, the end device goes back from class B to class A. The attack takes place when the whole network is dependent on a single gateway, which causes disruption between the end device and network server.

Butun et al. [7] made a security analysis of LoRaWAN v1.1 with the aim to review and clarify its security aspects. Their results of security risks are then analyzed and compiled into a list, which is ranked on impact and threat level. The most highlighted discovered threats are end device physical capture, rogue gateway, and self-replay attacks. The more relevant part of this paper is the analysis regarding the potential rogue gateway vulnerability and attacks they discovered.

Lin et al. [13] proposed an interesting solution based on blockchain technology to increase the trust value within the LoRaWAN network. It aims to build an open, trusted, decentralized, and tamper-proof system, which should be able to verify all the transactions that take place. Since LoRaWAN’s main aim is to be a low-cost network with a sufficiently adequate level of security, it is thus debatable whether the increase in cost with this kind of implementation would be the correct solution. Their proposed solution would introduce a lot of new elements, and their future work states that they will look to explore smart contract script technology to define an automated trading model or an automatic billing and roaming function.

The authors in [14] pointed out that in LoRaWAN, when the number of nodes joining the network server is large, high latency may occur in processing requests from all devices. To cope with this challenge, the authors proposed a multi-device authentication-based join mechanism. They considered the exclusive-OR technique for a large number of devices’ authentication. Furthermore, the hash operation is implied to protect the proposed technique against several threats such as session keys’ revealing, the end device. and network server’s attacks. The proposed technique outperforms and achieves 33% low latency as compared to the original LoRa specification.

Ribeiro et al. [15] proposed a secure architecture for key management in LoRaWAN. The proposed architecture is based on smart contracts and permission blockchain that enhances the security and availability of LoRaWAN infrastructure. The authors created a prototype using an open-source tool that achieves similar execution latency as compared to the traditional LoRaWAN system.

The authors in [16] discussed that the LoRaWAN join procedure needs security protection, as it is susceptible to multiple security issues. In addition, to cope with the security issues associated with the network server, the authors presented a lightweight two-factor authentication mechanism. Their method is based on blockchain technology that secures the LoRa joins procedure. This blockchain-based approach secures the LoRa join procedure by providing an extra layer of security. This approach is validated using the Ethereum blockchain and revealed that the proposed system achieved good throughput with the cost of low latency.

In [17], the authors proposed Ehpemeral Diffie–Hellman Over COSE (EDHOC) protocol for secure key provisioning in LoRaWAN, which is a lightweight protocol that provides a secure key establishment between the end device and network server. The EDHOC was used to derive the session keys, namely NwkSKey and AppSKey, which were used in the OTAA activation method. The proposed protocol can be supported by the spreading factors having the highest data rate i.e., SF7 and SF8.

In [8], Eldefrawy et al. considered that all GWs in the network are trusted entities which could not create a single point of failure (SPOF). Hence, their formal analysis did not reveal any problem related to GWs. On the contrary, in the current version of LoRaWAN, GWs are not authenticated by the servers or another Trusted Third Party (TTP). As we prove in this work, GWs can create an SPOF for attackers and from which they can execute network-based attacks, including hole attacks such as black-hole or selective-forwarding.

Haxhibeqiri et al. [18] presented an extensive analysis of LoRaWAN. The authors discussed the new version of LoRawAN v1.1 and came up with addressing several challenges and security vulnerabilities. However, the end-to-end MIC is missing, which makes payload integrity vulnerable. Furthermore, the node placed in the network can cause malicious activity between the network server and the application server. This malicious node leads the network to a bit-flipping attack.

The authors in [19] conducted a comprehensive survey for LoRaWAN architecture, applications, and security analyses. The authors stressed that promising enhancements have been counted in the LoRaWAN v1.1 standard such as improved communication and availability. However, the session duration is not mentioned by the new standard, which causes ambiguity regarding for how long the session will remain to continue. This needs to be addressed in the upcoming specification.

## 3. Various Aspects of LoRaWAN

### 3.1. Network Architecture

**Figure 1:**
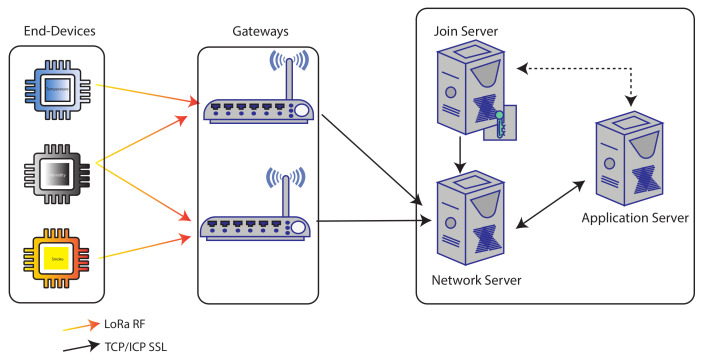
Network architecture of LoRaWAN.

The LoRaWAN network architecture can be described in five layers such as end device, gateway, network server, join server, and application server, as elucidated in Figure 1. The end device operates via radio frequency and forwards the packet to the gateway using dedicated frequency e.g., (433.05–434.79 MHz—Asia, 863–870 MHz—Europe, 902–928 MHz—US) [20]. The gateways are transparent and extend the packets received from the end device toward the network server. The gateway and network server communicate through TCP/IP protocol. The join server is a trusted entity and is used for the end device’s root keys distribution. The application server collects and sends the packets toward the network server, which is further transmitted to the end node through the gateway.

### 3.2. Communication

Gateways in LoRaWAN are tasked with demodulating LoRa packets using a packet forwarder, which forwards the packets to a LoRa NS. There exist several packet forwarders, most commonly: Message Queuing Telemetry Transport (MQTT) and Semtech User Datagram protocol (UDP).

- **Semtech UDP:** This forwarder was the first packet forwarder and still comes pre-compiled with most LoRa gateways. It uses the Semtech UDP protocol over TCP/UDP. Although over time, this protocol has acquired some flaws, it is still an easy way to test new gateways.
- **MQTT:** Is a lossless, bidirectional protocol designed for high-latency, low-bandwidth connections [21]. MQTT is a publish–subscribe protocol where clients subscribe to a set of topics for reading and writing. This makes the clients extremely lightweight and suitable for IoT connections, while the broker act as a gateway that handles all the transmissions to and from the servers of the relevant topics.

### 3.3. Message Format

The messages transferred over the network can be divided into two types; the first one is an uplink message which is sent from an end-device to the network, using the gateways as relays. The other type is called a downlink message, which is the opposite direction, from the server to an end-device using a gateway.

- **Uplink Message:** Uses the LoRa radio packet explicit mode, which consists of a physical header (PHDR) and a cyclic redundancy check (CRC) header (PHDR_CRC). Another CRC is required to protect the integrity of the payload; these three are together inserted by the radio transceiver in the following way:

Uplink PHY:PreamblePHDRPHDR_CRC
**PHYPayload**
CRC

- **Downlink Message:** Works very similar and also uses the LoRa radio packet explicit mode with a PHDR and a PHDR_CRC.

Downlink PHY:PreamblePHDRPHDR_CRC
**PHYPayload**


As can be seen above in either the uplink or downlink message, both contain a PHY payload called PHYPayload. This PHYPayload starts with a single-octet MAC header (MHDR), followed by a MAC payload (MACPayload) and finishing with a 4-octet message integrity code (MIC), as seen below:

PHYPayload:MHDR
**MACPayload**
MIC

Here, the MHDR specifies what message type it is (MType), there are six different ones. There is join request, join accept, unconfirmed data up/down and confirmed data up/down, where confirmed means that it has to be acknowledged by the receiver, while unconfirmed does not require that. The MACPayload carries information regarding the data frame.

MACPayload:FHDRFPortFRMPayload

The MACPayload contains a frame header (FHDR), which is the device address of an end-device (DevAddr), followed by an optional port field (FPort) and an optional frame payload field (FRMPayload) [22].

### 3.4. Security

The LoRaWAN specification consists of two layers of cryptography. The first is at the network level, which handles mutual authentication and integrity protection. The second layer is on the application level for confidentiality with end-to-end encryption [18].

The first layer consists of:

- **Mutual Authentication:** This is established between the end device and the LoRaWAN network during the join procedure, which ensures that both the device and the network are genuine and authentic.
- **Integrity Protection:** LoRaWAN MAC and application messaging are origin authenticated, integrity protected, replay protected, and encrypted. Together with the mutual authentication, it will protect the network by preventing the alteration of messages and ensure that the sender is legitimate.

In the second layer, we have:

- **Confidentiality:** For the application level, LoRaWAN employs end-to-end encryption for application packages that are transferred between an end device and application server.

These mechanisms use the AES algorithm to provide authentication and integrity of packets to the network server and end-to-end encryption to the application server. Each layer uses a unique 128-bit key, a network session key between the end device and network server, and an application session key for end-to-end on the application level. Through the use of two levels, it is able to achieve a “multi-tenant” shared network, where the network operator has no visibility on the payload data [23].

The two unique AES 128-bit session keys are:

- **Network Session Key:** (NwkSKey) is used as identification between the end-device and the network server.
- **Application Session Key:** (AppSKey) is for payload encryption and decryption and is shared end-to-end on the application level.

When an end device wants to access a network, it first has to be registered and then permitted to join the network. The corresponding keys can be generated in two ways:

- **Activation By Personalization** (ABP), this activation method already has the NwkSkey and AppSKey set up in advance and can thus access the network without requiring a join request.
- **Over-The-Air Activation** (OTAA), this method starts with a “Join Request” containing the device ID (DevEUI), the application server ID (AppEUI), and a random value called DevNonce [6]. It is signed with a message integrity code (MIC) using the AppKey. If the MIC is validated, then the node is authenticated, and the network sends back a “Join Accept” message, which is encrypted with the AppKey, and it includes the AppNonce and NetID parameters.

Both activation methods are viable; however, OTAA is preferred, since it can generate new keys every new session and also allows easy re-key if necessary [24]. Meanwhile, ABP is hardcoded and not as flexible, making it vulnerable to physical attacks.

## 4. Design and Implementation

**Figure 2:**
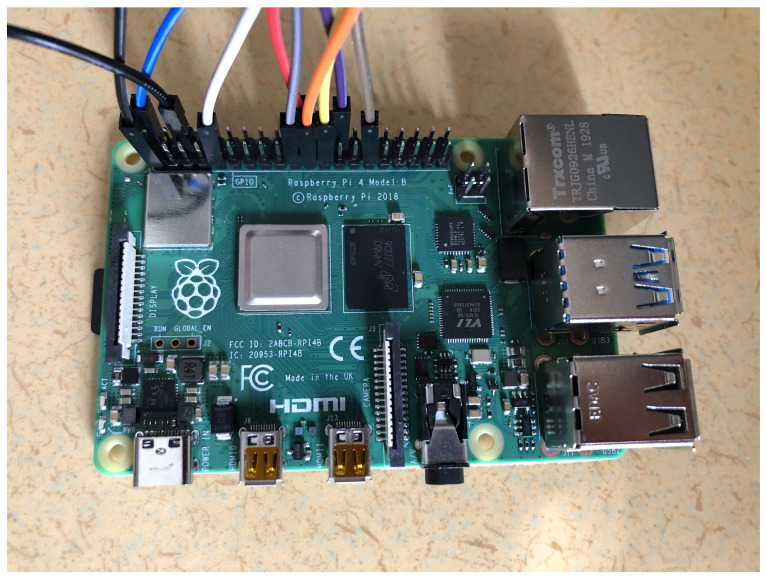
Computing unit—Raspberry Pi4 Model B.

This section discusses the practical demonstration of our proposed technique. The hardware that are used to replicate the LoRaWAN ecosystem, such as the end device and gateways, is done using a computing unit together with a LoRa radio chip. In this paper, the computational unit is the Raspberry Pi4 Model B (RPi4), as shown in Figure 2, with a 1.5 GHz quad-core processor and 4 GB RAM, together with complements such as a 16GB SD card, a breadboard, jumper cables to connect the different parts, and of course a power supply.

**Figure 3:**
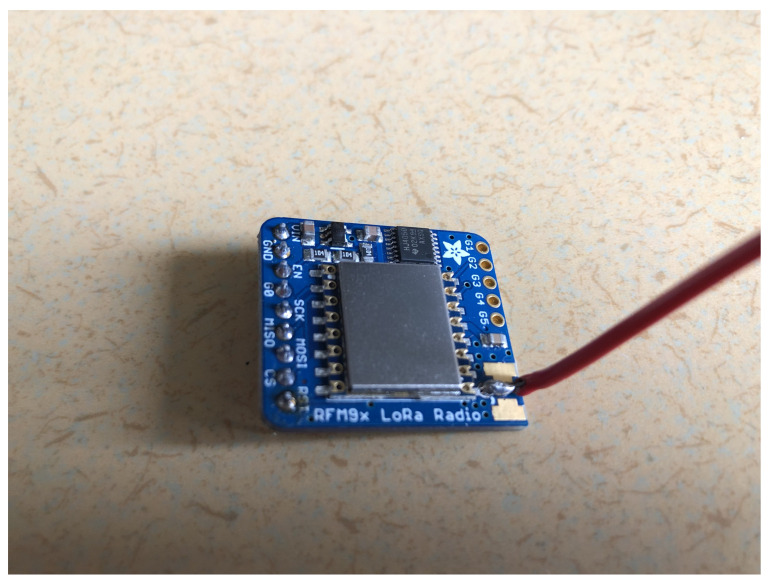
LoRa radio chip—Adafruit RFM96W.

The LoRa radio chip used is the Adafruit RFM96W LoRa Radio Transceiver 433 MHz, as shown in Figure 3, which is necessary in order to be able to use long-range communication in the LoRaWAN network. However, this chip is only able to provide a single channel at a time. A single channel is usually not enough for a LoRaWAN network and would cause a loss of messages, but for our experiments in a controlled environment, this is sufficient.

For the antenna, we used a simple 22 AWG wire, where the length of the wire determines its frequency using this formula:

(1) $$W_{l}=W_{v}\times F_{r}$$

where $W_{l}$ represents the wavelength, $W_{v}$ is the wave velocity, and $F_{r}$ is the radio frequency.

**Figure 4:**
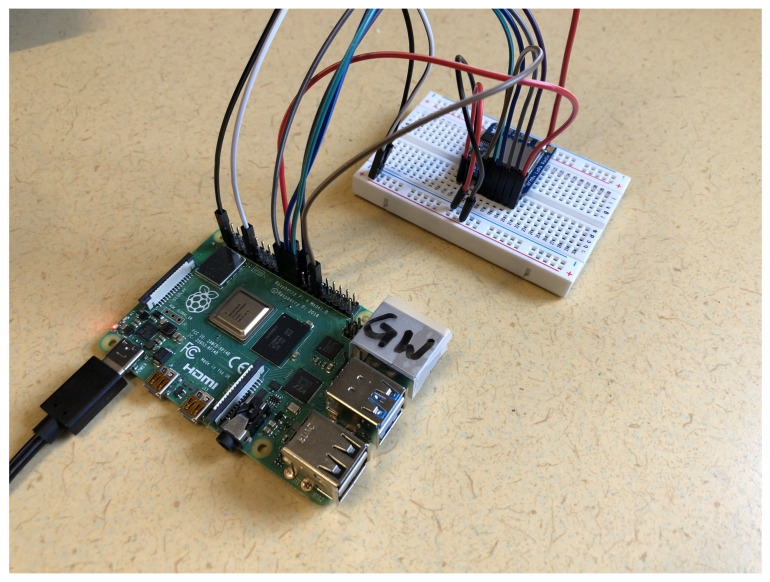
Gateway hardware and wiring setup using RPi4 and RFM96W.

**Figure 5:**
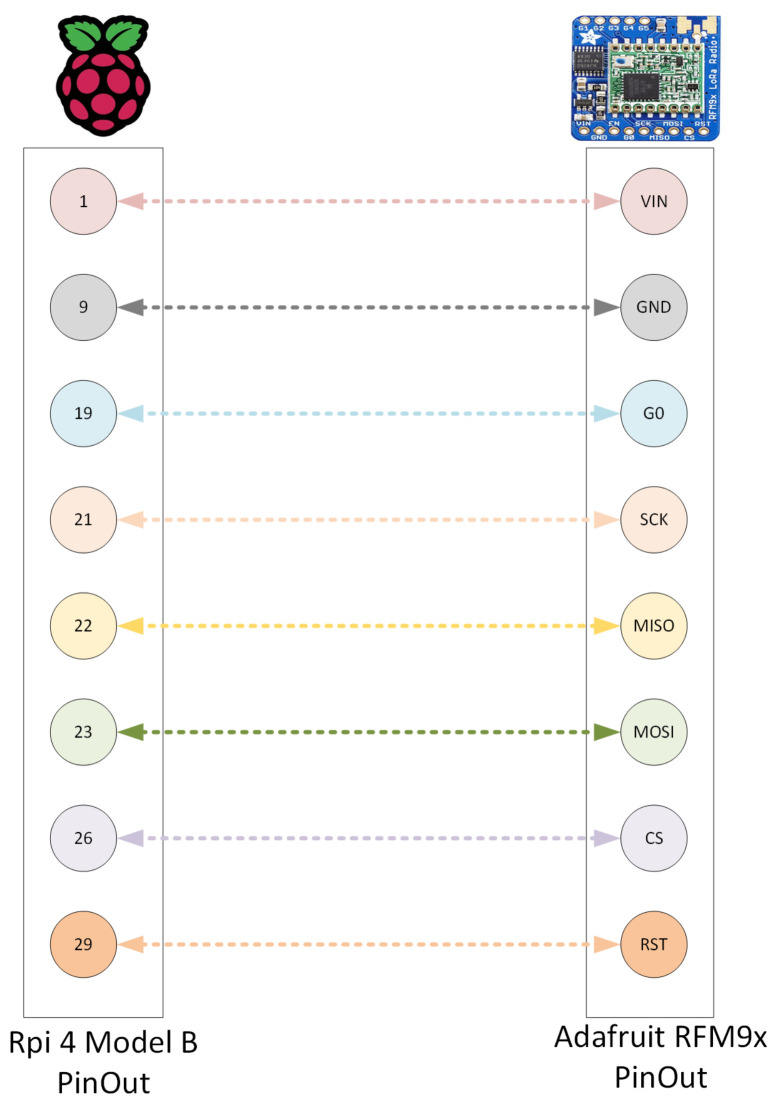
Wire cable connections between the Raspberry Pi and LoRa radio chip.

So, for the gateway, the computational unit and LoRa radio chip are connected. This was done by soldering the radio chip together with male header strip pins and then attaching it to a breadboard. When the chip is firmly set up on the breadboard, the breadboard is then wired with male-to-female wire cables to the Raspberry Pi, as seen in Figure 4 and the diagram in Figure 5. During our initial attempt, we did manage to set it up without the need of a breadboard; however, it did not feel sufficiently stable, and the wire antenna that was later soldered onto the chip could not be pointed upwards.

The LoRa radio chip consists of multiple pins that control different functions. Starting from the left, we have the three power pins: VIN, GND, and EN. These pins handle the powering of the breakout and shutdown of the radio.

- **VIN (Voltage Input):** The power supply can handle 3.3 to 6 VDC with a peak current of 150 mA, making sure to supply that amount of current for everything to work.
- **GND (Ground):** The ground is for logic and power.
- **EN (Enable):** The enable pin of the regulator, which is pulled high to VIN by default; pulling it low to GND will cut off the power to the radio.

The power pins are then followed by the six Serial Peripheral Interface (SPI) pins: G0, SCK, MISO, MOSI, CS, and RST. The SPI is a protocol that the microcontrollers use to communicate with peripheral devices.

- **G0 (GPIO 0/IRQ):** Is used for interrupt request notification from the radio to the microcontroller.
- **SCK (SPI Clock):** Is an input to the chip.
- **MISO (Master In Slave Out/Microcontroller In Serial Out):** Is for the data sent from the radio transceiver to the microcontroller/processor.
- **MOSI (Master Out Slave In/Microcontroller Out Serial In):** Is for the data sent from the micocontroller/processor to the radio transceiver.
- **CS (Chip Select):** Is an input to the chip. Drop it low to start an SPI transaction.
- **RST (Reset):** The reset pin is pulled high by default, which is reset. Pull it low to turn on the radio.

Similarly, the Raspberry Pi also comes with two rows of 40 General-Purpose Input/Output (GPIO) pins; out of these, we connect the radio chip to the eight equivalent pins with wire cables, as seen in Table 1.

### 4.1. Proposed Technique

OpenSSL software was installed on two Ubuntu 20.04.2 LTS in a virtual environment with two identical setups, except for the OpenSSL configuration files. Configuration files are necessary when using OpenSSL as a CA, as they contain more parameters than what is possible to specify in the terminal. OpenSSL configuration files provide two functions: template when issuing new certificates and enforce certificate policies in the configuration. A certificate policy contains a set of parameters such as countryName, commonName, etc., that must match corresponding fields in a Certificate Signing Request (CSR).

**Figure 6:**
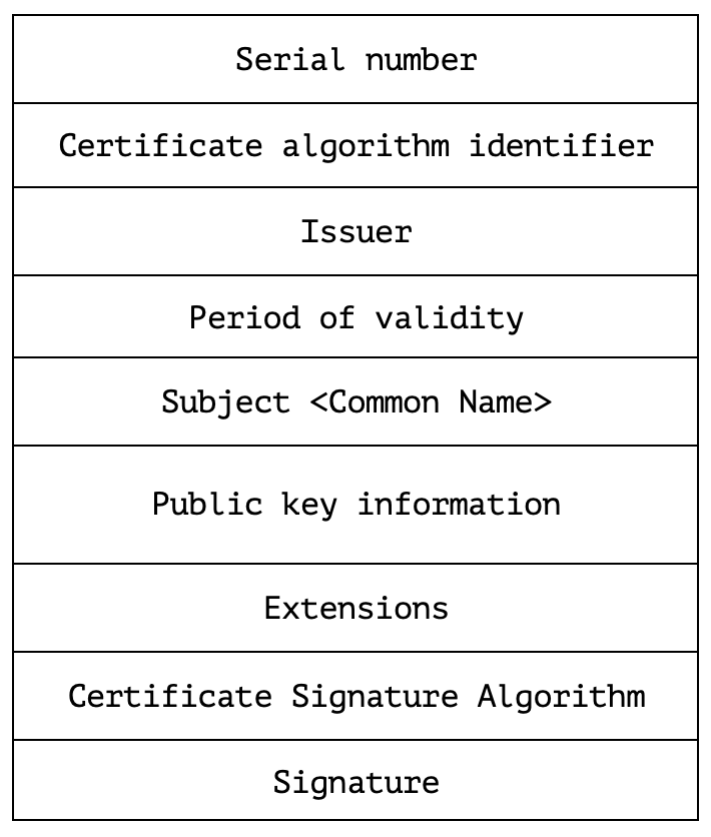
x.509v3 certificate format.

Figure 6 illustrates the x509v3-certificate format that the proposed solution will issue. The certificate format corresponds to the bare minimum required by the standard RFC for the purpose of reducing size. The attribute Common Name <CN> will hold the LoRaWAN unique device identifier DEVEUI; moreover, RSA will provide digital signatures.

To obtain a certificate, clients produce a CSR with their DEVEUI as the Subject Common <CN> and SHA256 as the message digest as seen in Listing 1.

**Listing 1.** Certificate Signing Request (CSR) for GW.

1


Certificate Request


2
    Data
3
          Version 1 (0x0)
4
          Subject : CN = 0xFFFFFFFFFFFF
5
          Subject Public Key Info :
6
              Public Key Algorithm : rsaEncryption
7
                  RSA Public - Key : (2048 bit )
8
                  Modulus :
9
                       00: af:e1 :3a:1a:d0 :7f:9c:c5:a9 :45:90:2 a:dc :88:
10
                       …
11
                  Exponent : 65537 (0 x10001 )
12
          Attributes :
13
          Requested Extensions :
14
              X509v3 Key Usage : critical Digital Signature , Key Encipherment
15
          X509v3 Extended Key Usage : E- mail Protection ,TLS Web Client
    Authentication
16
              X509v3 Subject Key Identifier :
17
                  67: FF :89:00:84: C7 :40: ED :54:33:05:74:75: DE:C1 :1E:4A :18:2 D:F4
18
              X509v3 Subject Alternative Name :
19
                  <EMPTY>
20


21
    Signature Algorithm : sha1WithRSAEncryption
22
          73: b4:c4:ed :93:9 e:f4 :9d:a7 :1f :90:40:71:07:5 d:3a:d9:f1:
23
          …

After this, the intermediate-CA issues a new certificate, which is illustrated by Listing 1, based on the CSR. The Certificate Revocation List (CRL) for the root-CA receives an update every seven days automatically or when a certificate is revoked. On the contrary, the CRL for the intermediate-CA receives an update every seven hours. The CRL is signed by the issuing entity and easily verified by recipients by comparing the signature with the signature generated from the issuing entity’s public key. Furthermore, the CRL from the issuing CA, i.e., intermediate-CA, will be placed beside the JS and accessed over MQTTv3 for LoRaWAN devices.

### 4.2. Attacker Model

**Figure 7:**
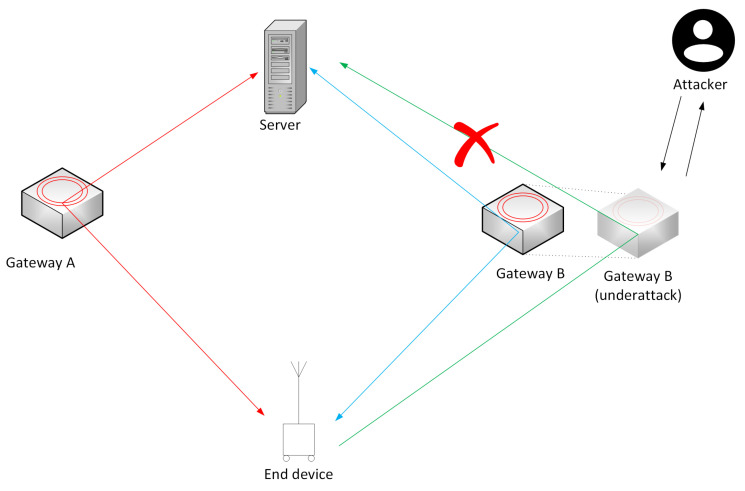
Scenario of packet advancement under MITM attack.

In this work, we are are aiming at devising a cyber-defense mechanism against GW-based attacks. In particular, Man-in-the-Middle (MITM) attacks are the focus of this work. This particular MITM attack is aimed at causing disturbances in availability. When LoRa packets are processed, the GW attaches information about itself for future downlink messages (may it be LoRa or application layer). Since the GWs are not verified, a potential rogue GW could attach malicious information such as the wrong sender IP, which causes future downlink messages not to get through. It is also possible that the rogue GW could attach correct metadata and then drop downlink messages, as shown in Figure 7. The network server (NS) must first ACK the LoRa-modulated packet forwarded by the rogue gateway to be efficient, since redundant messages are disregarded. Our proposal suggests to verify and authenticate GW <−−> NS, thus rendering these types of attack useless.

### 4.3. Verification of the Proposal

**Figure 8:**
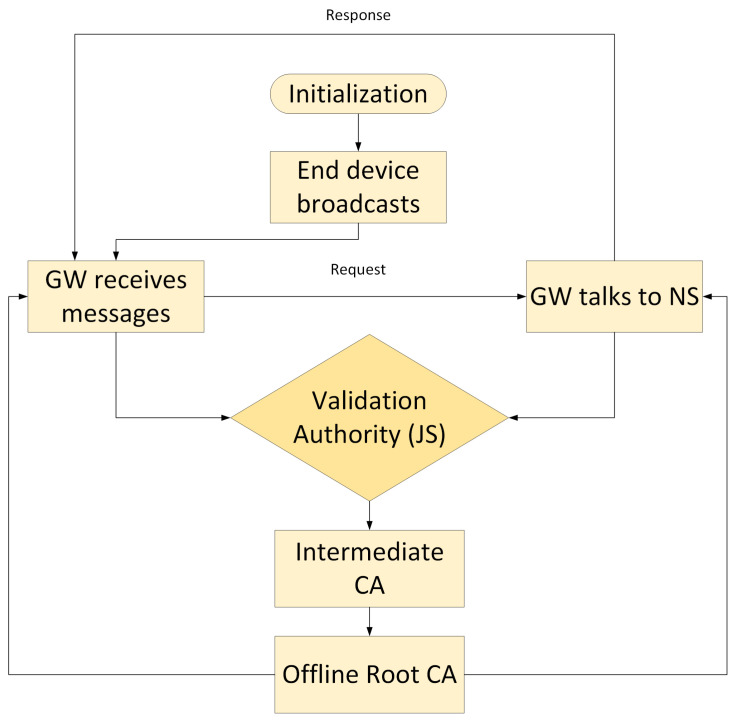
Flow chart of the certification process in the proposed work.

Extensive experiments have been carried out in order to verify the solution. The goal of each of the tests is to see how the proposed solution, as illustrated in Figure 8, handles unauthorized transmissions. As can be seen in the flow chart, the JS receives requests from both the GW and NS and forwards them to the intermediate-CA. The root-CA then authenticates requests and provides acknowledgment to the GW and NS.

This will be performed by collecting the Package Delivery Ratio (PDR) for each test scenario. The PDR is a metric used to show the percentage of packages that arrives out of the total number of packages sent in a network. Observing the PDR shows what happens when the network is either attacked by malicious attacks or when it is protected by the proposed solution and how that affects the network traffic.

(2) $$PDR=\frac{P_{Received}}{P_{Sent}}$$

A way to verify that our proposed solution is working would be to try and replicate a rogue gateway attack (executed as MITM attack), as seen in Figure 7. The attack that will be studied closely is the Selective Forwarding Attack, which exploits the frame counter. So, when an end-device would send out a certain amount of packages, these packages are registered in order and increments the frame counter. In other words, each device keeps track on all the messages it receives and thus only accepts messages with a larger frame counter. So, if it receives a message with a lower frame counter, then it is discarded. So, with Selective Forwarding Attack, a rogue actor could then withhold the majority of packages as long as it sends messages with a higher frame counter then the previous one, for example, just sending the first and the last package. Thus, if the rogue actor is faster than legitimate ones, either by being closer or outright disabling legitimate GWs in the vicinity, that would mean it could control the transmission flow and greatly reduce the network’s efficiency.

This can be validated by deploying two gateways, where one is an authorized gateway, GW-A, and the other is not, GW-B. Then, an end-device transmits a series of messages that will be picked up and forwarded to the NS. Starting first in the vicinity of the authorized gateway, GW-A, the majority of packages will first go through GW-A before arriving at the NS, where they all would ultimately be accepted. By then, we slowly move the ED closer to the rogue gateway, GW-B, and messages should at a certain point start to arrive first at the GW-B. If then a number of messages manage to arrive first at the GW-B before reaching the legitimate GW-A, the rogue gateway can then drop some packages and send one with a higher frame counter. The network server will just see an acceptable frame counter, which is higher than the previous one and accept the transmission. However, when the packages from GW-A finally arrive, they will come in order with a lower frame counter, which the NS ultimately rejects for being duplicates, which in the end means that the NS would only receive a fraction of the whole message.

## 5. Results

This section presents the results obtained from the verification and validation process; additionally, it presents the effectiveness of the proposed protocol for mutual authentication against attacks involving rogue gateways, as described by Butun et al. [7]. The verification process tests, and by extension determines, if the proposed protocol meets its specification for mutual authentication. Moreover, the verification process determines the effectiveness against rogue gateways attacks.

### RSSI of the Test Area

The Received Signal Strength Indicator (RSSI) is a parameter aimed to mean how well a device can hear, detect, and receive transmissions. This is based on the relative quality of the signal and any potential loss due to the antenna or cable properties. The RSSI may be reported in many different ways, but a common method adopts decibel-milliWatts (dBm), for example the authors in [25] report indoor localization experiments based on RSSI evaluation, adopting a matching schema such as the one detailed in Table 2.

By measuring the RSSI, data can be gathered that would give a rough estimate of the noise levels, which could affect the signal strength. This is valuable information that would give the reader sufficient insight in the RSSI of urban areas and to help understand the baseline scenario. It would also verify how solid the hardware setups performance is, as there were some concerns on the signal strength of the makeshift antenna.

**Figure 9:**
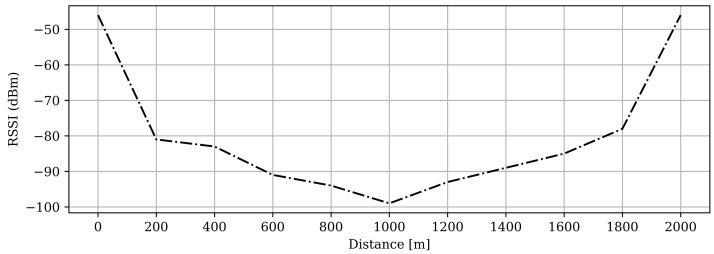
The RSSI values of the baseline scenario, the first half being the RSSI of GW-A and the second half GW-B.

Figure 9 shows the same baseline scenario with the RSSI values collected and then plotted. As can be seen, the RSSI already starts past the −80 dBm value for the initial distance, which is a low rating according to Table 2. This continues to deteriorate as the end-device travels further away. At around 1000 m, there are no longer any packages being delivered to GW-A, which correlates to an RSSI value almost reaching −100 dBm, which seems reasonable from the literature study. A similar trend can be seen for GW-B: as the ED gets closer, the RSSI strength increases.

Let us consider the implications of a Selective Forwarding Attack in critical infrastructure. Let us examine water height meters for flood embankments in the context of the smart city.

**Figure 10:**
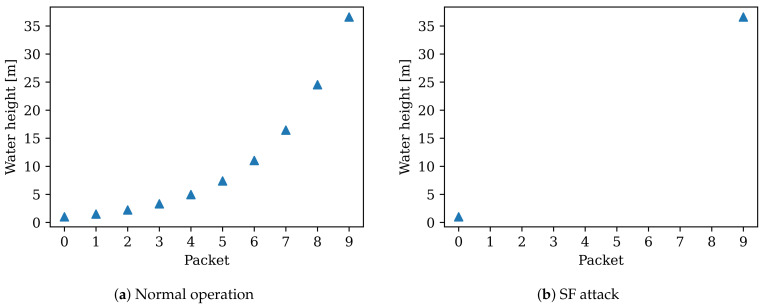
(**a**) Water height values (indicated by triangles) gathered from end-device with no attack present, (**b**) water height values with a Selective Forwarding Attack present.

Figure 10 depicts (a) a plot or potential dashboard over water height measurements during nine packets, whereas (b) shows the water height measurements during a Selective Forwarding Attack. In Figure 10a, the water level rises incrementally. Meanwhile, Figure 10b shows water rising from 1 m to over 35 m in two readings. If the system is autonomous, it may decide to open the flood gates and cause harm to the people and buildings in the immediate area. If the system instead is monitored by humans, a tedious and time-consuming task will take place to determine the fault of the problem.

By using the low-cost and accessible RFM9x LoRa transceivers combined with the Raspberry Pi, our gateways practically became single-channel. Single-channel gateways can only receive payload on a specific spreading factor and channel. Additionally, single-channel gateways offer reduced coverage compared to higher-end LoRa chips [26]. In the baseline measurements, we noted a drastically reduced PDR after 200 m, in a moderate Line of Sight. After 1000 m, we did not receive any packets. Furthermore, we had only one end-device at our disposal, which greatly affected the type of setups we could simulate. The RSSI readings obtained from the RFM9x, which are crucial for determining the signal strength and quality, are not that accurate. It is also important to note that single-channel gateways are not LoRaWAN-compliant for the reasons stated above.

**Figure 11:**
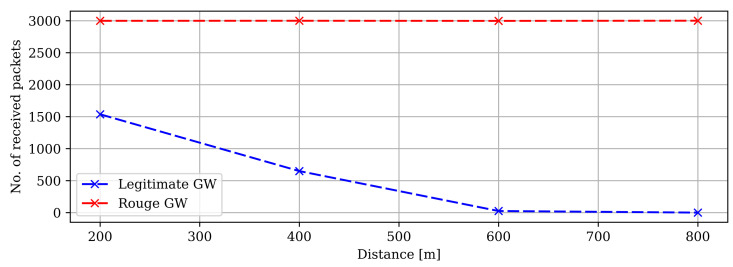
Preliminary testing area with no line of sight. R-GW permanently placed at 200 m from the ED and L-GW placed at 200 m increments up to 800 m.

**Figure 12:**
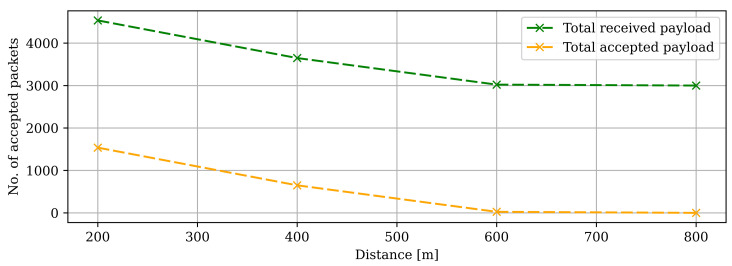
Preliminary testing area with no line of sight. Data on packages received by the NS, total vs. accepted.

Figure 11 showcases the results obtained from the proposed technique. As can be seen, the rogue gateway receives a higher number of packets than the legitimate gateway. It is because of the fixed position near the end device. Therefore, the rogue gateway instantly received the packets when broadcast from the end device. In other words, the legitimate gateway received fewer packets as of a long distance from the end device. The plot in Figure 12 shows the number of packets received by the network server. At 200 m, the network server received more than 4500 packets. However, due to our novel authentication algorithm, only the packets from the legitimate gateway were accepted (approx. 1500 packets), and the rest were discarded (approx. 3000 packets) by the network server.

## 6. Discussion

LoRaWAN is an emergent technology that provides the connectivity facility over long distances with ultra-low power consumption. It brings a strong security feature as the AES-128 encryption technique that is used for the payload travels from the end device to the application server. However, previous research studies have noted that the gateway is the weak point, which provides an opportunity for attackers in the network. As discussed earlier, the whole network consists of end devices, gateways, and servers. The gateway plays the key role that collects the messages from the end devices and transmits the collected messages to the servers. The overall network relies on the gateway, and the malicious attacks can affect the network. To cope with malicious attacks, we present a novel certificate authentication technique to protect the gateway in the network. The proposed technique considers a PKI solution that consists of Certification Authority (CA), Registration Authority (RA), Validation Authority (VA), and key pairs. For security reasons, we kept the root-CA offline and used an intermediate certificate signed by root-CA.

Moreover, ongoing research studies identified several vulnerabilities at LoRaWAN that circumvent the strong encryption process (the AES-128). As a solution, many researchers have introduced implicit certificates (such as Elliptic Curve Qu-Vanstone—ECQV) for authentication between nodes and the Application Server. These are cumbersome, to say the least. ECQV works by reconstructing values derived from a shared public key. ECQV does not support certificate revocation; hence, a physically compromised GW can be used to attack the network. Secondly, our proposal is overall best suited for time and security critical applications such as large enterprises, etc. Table 3 summarizes and compares unique features of related security solutions for LoRaWAN in the literature vs. our proposal.

## 7. Conclusions and Future Work

LoRaWAN is a promising technology that enables long-range communication with extremely low power consumption. Gateways are one of the most important elements in LoRaWAN, in the case: “deal” with the transportation of packets from the end device to the network server and vice versa. However, recent studies have pointed out that the gateway constitutes the weakest point in the network and is susceptible to a variety of malicious attacks. Therefore, this paper proposes the latest technique to prevent the gateway from malicious attacks such as Selective Forwarding Attack. The proposed technique considers a PKI-solution that considers a two-tier CA setup, i.e., a root-CA and intermediate-CA. The simulation results reveal the effectiveness of the proposed work. In the future, a lightweight certificate-assigning technique might reduce the payload size during initial authentication.

According to our experimentation, 66.67% of the packets that are arriving from an illegitimate GW are discarded in our devised secure and reliable solution.

### Future Work

Future research may find a solution for reducing the certificate size that might be beneficial for the network server to keep the certificates for all authenticated gateways in the memory. Such small sizes can also help the throughput when many gateways are authenticated and forwarding payloads.

## Figures and Tables

**Table 1:** Wire connection of the pins.

Raspberry Pi 4	RFM96W
1 (3V3 Power)	VIN
9 (Ground)	GND
29 (GPIO 5)	G0
23 (GPIO 11: SCLK)	SCK
21 (GPIO 9: MISO)	MISO
19 (GPIO 10: MOSI)	MOSI
26 (GPIO 7:CE1)	CS
22 (GPIO 25)	RST

**Table 2:** Signal strength levels of RSSI [25].

Signal Strength	Rating	Info
>−30 dBm	Amazing	Max signal strength, due to being right next
		to the client. Not reasonable in the real world.
−50 dBm	Excellent	Almost perfect signal strength in the real world
		with ideal conditions.
−60 dBm	Very Good	High latency, would most likely not feel any
		disturbance.
−70 dBm	Good	Minimum signal strength for reliable packet
		delivery for menial tasks.
−80 dBm	Low	Minimum signal strength for basic connectivity.
		Packet delivery is now unreliable.
−90 dBm	Very Low	Terrible signal strength, with frequent package
		drops and connectivity issues.
<−100 dBm	No Signal	Not much if anything is able to get through.

**Table 3:** Literature comparison of related security solutions for LoRaWAN.

Related Work	Authentication of End-Device with Server	Improvements on End-Device Comm	Improvements on Network Security	Authentication of GW with Server
Mårlind and Butun [4]	✔	✔	✔	✖
Gresak and Voznak [9]	✖	■	✔	✖
Fan et al. [14]	✔	✔	■	✖
Ribeiro et al. [15]	✔	✔	✔	✖
Danish et al. [16]	✔	✔	■	✖
Sanchez et al. [17]	✔	✔	✔	✖
Naoui et al. [27]	✖	■	✔	✖
**Proposed work**	✖	✔	✔	✔

**Legend**✖: Does not fulfill; ✔: Fulfills; ■: Inclusive.

## Data Availability

Not applicable.

## Abbreviations

The following abbreviations are used in this manuscript:

AS	Application Server
AppSKey	Application Session Key
ABP	Activation By Personalization
CA	Certificate Authority
CRC	Cyclic Redundancy Check
CRL	Certificate Revocation List
CSS	Chirp Spread Spectrum
CSR	Certificate Signing Request
CTR	Counter
dBm	decibel-milliWatts
ECQV	Elliptic Curve Qu-Vanstone
EDHOC	Ehpemeral Diffie–Hellman Over COSE
GW	Gateway
HDR	Frame Header
IoT	Internet of Things
ISM	Industrial Scientific and Medical
JS	Join Server
LoRa	Long Range
LoRaWAN	Long Range Wide Area Network
MQTT	Message Queuing Telemetry Transport
MITM	Man-in-the-Middle
MTyope	Message Type
MIC	Message Integrity Code
M2M	Machine to Machine
NwkSKey	Network Session Key
NS	Network Server
OTAA	Over-The-Air Activation
PDR	Packet Delivery Ratio
PKI	Public Key Infrastructure
RA	Registration Authority
RF	Radio Frequency
RSSI	Received Signal Strength Indicator
SPOF	Single Point of Failure
TTP	Trusted Third Party
VA	Validation Authority
